# SLAM Project - Long Term Ecological Study of the Impacts of Climate Change in the natural forest of Azores: IV - The spiders of Terceira and Pico Islands (2019-2021) and general diversity patterns after ten years of sampling

**DOI:** 10.3897/BDJ.10.e96442

**Published:** 2022-11-04

**Authors:** Sébastien Lhoumeau, Pedro Cardoso, Ricardo Costa, Mário Boieiro, Jagoba Malumbres-Olarte, Isabel R. Amorim, François Rigal, Ana M. C. Santos, Rosalina Gabriel, Paulo A. V. Borges

**Affiliations:** 1 cE3c- Centre for Ecology, Evolution and Environmental Changes, Azorean Biodiversity Group, CHANGE – Global Change and Sustainability Institute, Faculty of Agricultural Sciences and Environment, University of the Azores, Rua Capitão João d´Ávila, Pico da Urze, 9700-042, Angra do Heroísmo, Azores, Portugal cE3c- Centre for Ecology, Evolution and Environmental Changes, Azorean Biodiversity Group, CHANGE – Global Change and Sustainability Institute, Faculty of Agricultural Sciences and Environment, University of the Azores, Rua Capitão João d´Ávila, Pico da Urze, 9700-042 Angra do Heroísmo, Azores Portugal; 2 LIBRe – Laboratory for Integrative Biodiversity Research, Finnish Museum of Natural History, University of Helsinki, P.O.Box 17 (Pohjoinen Rautatiekatu 13), 00014, Helsinki, Finland LIBRe – Laboratory for Integrative Biodiversity Research, Finnish Museum of Natural History, University of Helsinki, P.O.Box 17 (Pohjoinen Rautatiekatu 13), 00014 Helsinki Finland; 3 IUCN SSC Mid-Atlantic Islands Specialist Group, Angra do Heroísmo, Azores, Portugal IUCN SSC Mid-Atlantic Islands Specialist Group Angra do Heroísmo, Azores Portugal; 4 Institut Des Sciences Analytiques et de Physico Chimie pour L’environnement et les Materiaux UMR5254, Comité National de la Recherche Scientifique - University de Pau et des Pays de l’Adour - E2S UPPA, Pau, France Institut Des Sciences Analytiques et de Physico Chimie pour L’environnement et les Materiaux UMR5254, Comité National de la Recherche Scientifique - University de Pau et des Pays de l’Adour - E2S UPPA Pau France; 5 Terrestrial Ecology Group (TEG-UAM), Departamento de Ecología, Universidad Autónoma de Madrid, 28049, Madrid, Spain Terrestrial Ecology Group (TEG-UAM), Departamento de Ecología, Universidad Autónoma de Madrid, 28049 Madrid Spain; 6 Centro de Investigación en Biodiversidad y Cambio Global (CIBC-UAM), Universidad Autónoma de Madrid, 28049, Madrid, Spain Centro de Investigación en Biodiversidad y Cambio Global (CIBC-UAM), Universidad Autónoma de Madrid, 28049 Madrid Spain

**Keywords:** Arthropoda, Araneae, long-term sampling, Azores, Macaronesia, native forest, SLAM trap

## Abstract

**Background:**

Long-term studies are key to understand the drivers of biodiversity erosion, such as land-use change and habitat degradation, climate change, invasive species or pollution. The long-term project SLAM (Long Term Ecological Study of the Impacts of Climate Change in the natural forest of Azores) started in 2012 and focuses on arthropod monitoring, using SLAM (Sea, Land and Air Malaise) traps, aiming to understand the impact of the drivers of biodiversity erosion on Azorean native forests (Azores, Portugal). This is the fourth contribution including SLAM project data and the second focused on the spider fauna (Arachnida, Araneae) of native forests on two islands (Pico and Terceira). In this contribution, we describe data collected between 2019 and 2021 and we analyse them together with a previously published database that covered the 2012-2019 period, in order to describe changes in species abundance patterns over the last ten years.

**New information:**

We present abundance data of Azorean spider species for the 2019-2021 period in two Azorean Islands (Terceira and Pico). We also present analyses of species distribution and abundance of the whole sampling period. In the period of 2019-2021, we collected a total of 5110 spider specimens, of which 2449 (48%) were adults. Most juveniles, with the exception of some exotic Erigoninae, were also included in the data presented in this paper, since the low diversity of spiders in the Azores allows a relatively precise species-level identification of this life-stage. We recorded a total of 45 species, belonging to 39 genera and 16 families. The ten most abundant species were composed mostly of endemic or native non-endemic species and only two exotic species (*Tenuiphantestenuis* (Blackwall, 1852) and *Dysderacrocata* C. L. Koch, 1838). They included 4308 individuals (84%) of all sampled specimens and were the dominant species in Azorean native forests. The family Linyphiidae was the richest and most abundant *taxon*, with 15 (33%) species and 2630 (51%) specimens. We report *Cheiracanthiummildei* L. Koch, 1864, a non-native species, from Pico Island for the first time. We found no new species records on Terceira Island. This publication contributes to increasing the baseline information for future long-term comparisons of the spiders on the studied sites and the knowledge of the arachnofauna of the native forests of Terceira and Pico, in terms of species abundance, distribution and diversity across seasons for a 10 years period.

## Introduction

Humanity is facing a biodiversity crisis (Caujapé-Castells et al. 2010, Hanski 2011, Heleno et al. 2020, Fernández-Palacios et al. 2021) due to recent expansion and intensification of human disturbance, which is particularly visible on island systems (Baret et al. 2006, Gaspar et al. 2008, Borges et al. 2019, Borges et al. 2020). The spread of exotic species is one of the major concerns, as biotic invasions are recognised as one of the main causes of species extinction (Pyšek et al. 2020). Scientists have pointed out the critical importance to consider long-term monitoring schemes to track species’ response to introductions for a better assessment of extinction risk. However, long-term baseline studies are the exception rather than the rule in literature, especially for less conspicuous taxa, such as arthropods (Henriques et al. 2017, Borges et al. 2018, Costa and Borges 2021, Vancutsem et al. 2021). Although long-term monitoring may be expensive and/or hard to implement (Caughlan and Oakley 2001), the resulting data allow the recording of changes in biodiversity composition and abundance, which allow for better estimates of the trends and more accurate predictions of future of communities through the so-called time series analyses (Loh et al. 2005, Dornelas et al. 2013, Coops et al. 2014, Dornelas et al. 2014) and is, thus, well worth the effort.

Spiders are one of the most well-known groups of arthropods in the Azores Archipelago due to a number of past studies (see, for example, Borges and Wunderlich 2008, Cardoso et al. 2010, Borges et al. 2013, Malumbres-Olarte et al. 2019, Carvalho et al. 2021) and they are one of the most useful to assess biodiversity change (New 1999). Nevertheless, we still lack suitable population and demographic information, which limits proper assessment of the conservation status of many species and of the colonisation dynamics of alien species that threaten the ecosystem (Borges et al. 2020).

Since 2012, we have sampled the arthropod communities in the remaining native forests fragments of the Azores Archipelago through the SLAM project (Long Term Ecological Study of the Impacts of Climate Change in the natural forest of Azores), using a large number of SLAM traps across several islands (Borges et al. 2017, Matthews et al. 2018, Costa and Borges 2021, de Vries et al. 2021, Tsafack et al. 2021, Borges et al. 2022a, Borges et al. 2022b).

## General description

### Purpose

This publication is the fourth data-paper contribution to the long-term project SLAM (Long Term Ecological Study of the Impacts of Climate Change in the natural forest of Azores) that started in 2012 with the aim of understanding the impact of the drivers of biodiversity erosion on Azorean native forests (Azores, Portugal) (see Costa and Borges 2021, Borges et al. 2022a, Borges et al. 2022b). This publication is also the second of the series that explores time-series data for the spider fauna in Pico and Terceira Islands (Azores Archipelago) (see the first contribution in Costa and Borges 2021).

**Figure 1.**
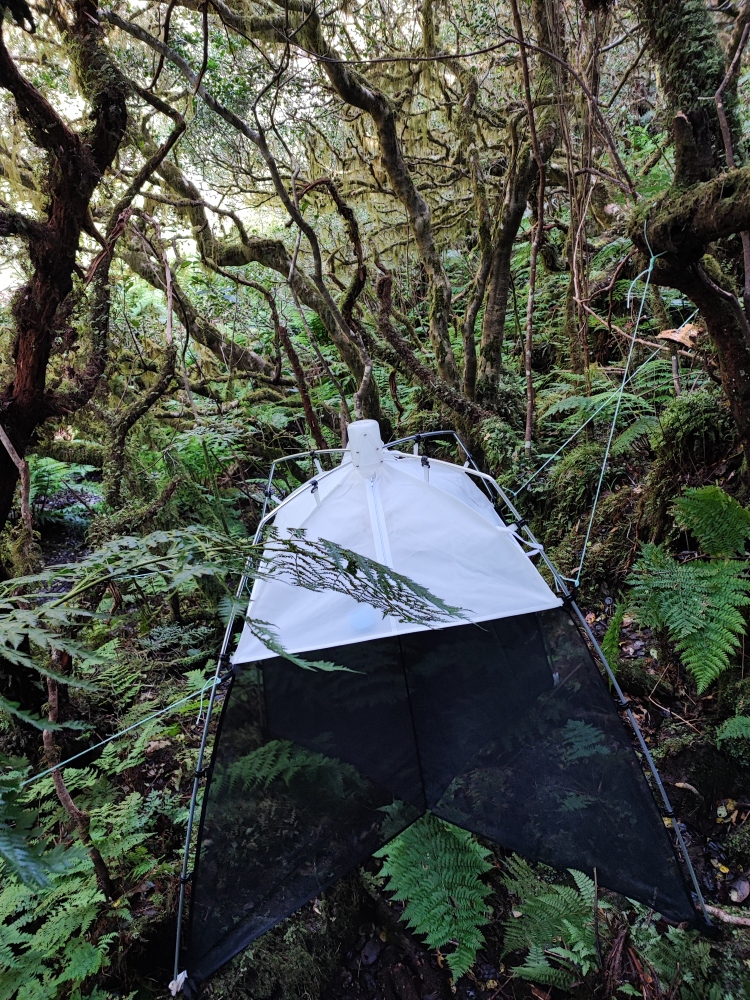
SLAM - Sea, Land and Air Malaise trap. Credit: Paulo A. V. Borges.

We used passive flight interception SLAM traps (Sea, Land and Air Malaise trap) (MegaView Science Co. Ltd., Taichung City, Taiwan) (Fig. 1) to sample native forest plots in several Azorean islands, with one trap placed at each plot. This publication aims to document the most recent data (from winter 2020 to autumn 2021 on Terceira and from winter 2019 to Autumn 2021 on Pico) as an extension of the previous database published by Costa and Borges (2021) following the same sampling strategy. Besides, we also incorporate a recent taxonomic change over the database, where *Sancusacoreensis* (Wunderlich, 1992) becomes *Leucognathaacoreensis* Wunderlich, 1992 (Ceccolini and Cianferoni 2022).

### Additional information

The year 2012 marks the beginning of the survey of arthropods in Terceira Island through SLAM traps, within the Project NETBIOME ISLANDBIODIV. In Pico Island, the study started in September 2013. Since 2020, the SLAM project has been financed within the project LIFE-BEETLES. Samples were collected by the University of the Azores team members in Terceira Island and by Pico Nature Park rangers in Pico Island.

The statistical analyses presented and commented in the Discussion are based on the complete dataset on spiders from 2012-2021, including all samples since the beginning of the study, using also the data published by Costa and Borges (2021). In a few cases, it was impossible to collect the SLAM traps on some sites and seasons (22 EventID out of 665 recorded) and sampling periods extended over three months. In order to be consistent, we removed sampling events that are related to more than one season.

## Project description

### Title

SLAM - Long Term Ecological Study of the Impacts of Climate Change in the natural forest of Azores

### Personnel

Paulo A.V. Borges conceived and coordinated the project.

**Fieldwork**: For the period 2019-2021 (Terceira Island) - Paulo A. V. Borges, Rui Carvalho, Rui Nunes, Sébastien Lhoumeau; (Pico Island) - Paulo Freitas, Sónia Manso.

**Parataxonomists**: For the period 2019-2021 – Abrão Leite, Adrian Fernandez Marinez, Emanuela Cosma, Jonne Bonnet, Joel Martin Aye, Loïc Navarro, Magí Ramon Martorell, Marco Canino, Natalia Fierro Frerot, Sébastien Lhoumeau, Valentin Moley.

**Taxonomists**: Paulo A. V. Borges and Luís Carlos Crespo

**Curation**: Voucher specimen management was mainly undertaken by Abrão Leite, Sébastien Lhoumeau and Paulo A. V. Borges.

### Study area description

**Figure 2.**
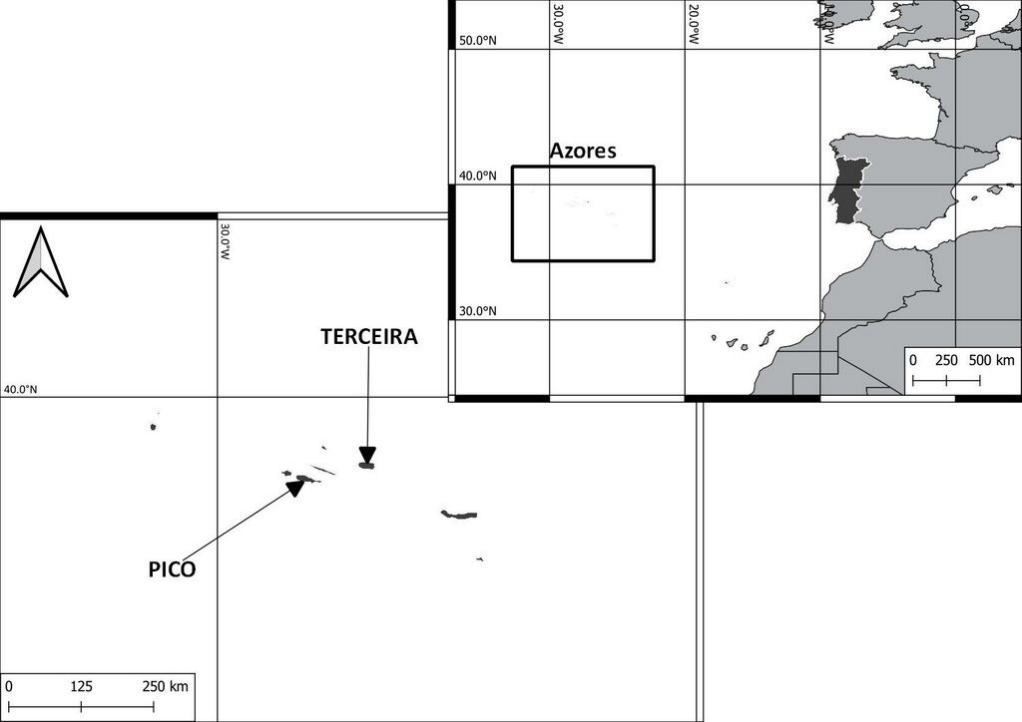
Location of the Azores Archipelago and the Islands of Pico and Terceira.

**Figure 3.**
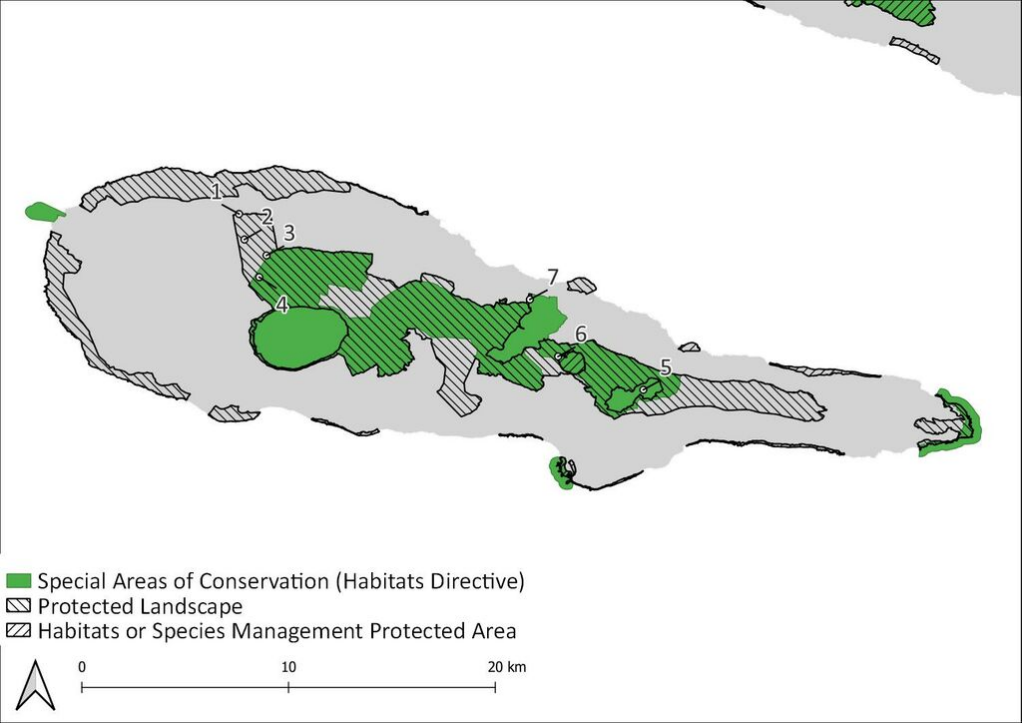
Location of sampling sites on Pico Island 1: PIC_ML_200; 2: PIC_ML_400; 3: PIC_ML_600; 4: PIC_ML_800; 5: PIC-NFCA-T-09; 6: PIC-NFLC-T-02; 7: PIC-NFMP-T-03.

**Figure 4.**
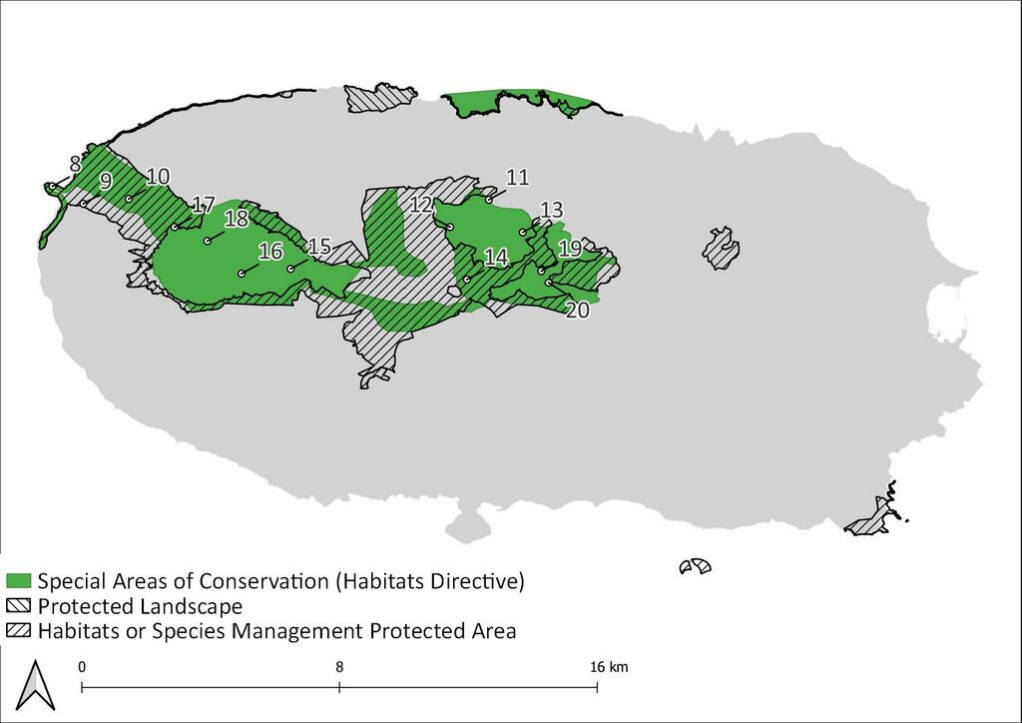
Location of sampling sites on Terceira Island. 8: TER_0M; 9: TER_200M; 10: TER_400M; 11: TER-NFBF-T-01; 12: TER-NFBF-T-02; 13: TER-NFBF-TP41; 14: TER-NFPG-T-33; 15: TER-NFSB-T-07; 16: TER-NFSB-T164; 17: TER-NFSB-TE48; 18: TER-NFSB-TE49; 19: TER-NFTB-T-15; 20: TER-NFTB-T-18_Original.

The Azores are an isolated archipelago (38°43′49″N, 27°19′10″W, Fig. 2), situated in the mid-Atlantic Ocean comprising nine volcanic Islands spread over 500 km in a W/NW–E/SE direction. During this project, eight Islands (Corvo, Flores, Faial, Pico, Graciosa, Terceira, S. Miguel and S. Maria) were surveyed within the SLAM Project. However, only Pico (Fig. 3) and Terceira Islands (Fig. 4) were continuously monitored since 2012 and 2013, respectively and are, thus, selected for this work.

### Design description

We sampled in the Azorean Islands of Terceira and Pico, four times per year (mid-March (winter sample), mid-June (spring sample), mid-September (summer sample) and mid-December (autumn sample)).

### Funding

The following sources of funding were available during the 2019-2021 period:

- FEDER - AZORESBIOPORTAL –PORBIOTA (ACORES-01-0145-FEDER-000072)

- EU ERASMUS + Training Grants to Adrian Fernandez Marinez, Emanuela Cosma, Jonne Bonnet, Joel Martin Aye, Loïc Navarro, Magí Ramon Martorell, Marco Canino, Natalia Fierro Frerot, Sébastien Lhoumeau and Valentin Moley.

- Direcção Regional do Ambiente – LIFE-BETTLES (LIFE18 NAT_PT_000864).

- Science and Technology Foundation (FCT) - MACRISK-Trait-based prediction of extinction risk and invasiveness for Northern Macaronesian arthropods (FCT-PTDC/BIA-CBI/0625/2021).

- Portal da Biodiversidade dos Açores (2022-2023) - PO Azores Project - M1.1.A/INFRAEST CIENT/001/2022.

## Sampling methods

### Study extent

Overall, we sampled a total of twenty plots, thirteen on Terceira Island and seven on Pico Island, using SLAM traps (Table 1) (see Costa and Borges 2021). The plots are located in some of the best preserved native forest patches of the two Islands, having only limited human disturbance (Borges et al. 2017).

The sampling plots are mostly dominated by endemic vegetation like *Juniperusbrevifolia*, *Ericaazorica*, *Laurusazorica* and *Ilexazorica* (see Borges et al. 2017 for more details). In Pico Island, the plots located at lower elevations (0-400 m a.s.l.) are dominated by *Ericaazorica* and *Morellafaya*, but with some presence of the invasive species, *Pittosporumundulatum*. At higher elevations (600-1000 m a.s.l.), the dominant vegetation is similar to that found in Terceira Island’ plots.

### Sampling description

In the laboratory, specimen sorting and spider identification followed standard procedures, using morphologic and copulatory features for species identification. A reference collection was made for all collected specimens (whether or not identified at species level) by assigning them a morphospecies code and depositing them at the Dalberto Teixeira Pombo Insect Collection (DTP), University of Azores (Terceira Island).

Spider juvenile identification is very important in spider studies (Domènech et al. 2022). Most juveniles, with the exception of some exotic Erigoninae, were also included in the data presented in this paper, since the low diversity of spiders in the Azores allows a relatively precise species-level identification of this life-stage.

## Geographic coverage

### Description

Pico and Terceira Islands, the Azores, Macaronesia, Portugal (Fig. 2)

### Coordinates

38.835 and 38.372 Latitude; -28.592 and -26.993 Longitude.

## Taxonomic coverage

### Description

Araneae (Arthropoda, Arachnida)

## Traits coverage

Functional trait data including detailed morphometric measurements for most of the studied species can be accessed in the publication by Macías-Hernández et al. (2020).

## Temporal coverage

### Notes

11 December 2019 to 12 March 2022 for Terceira Island and 17 December 2018 to 7 January 2022 for Pico Island.

## Collection data

### Collection name

Dalberto Teixeira Pombo insect collection at the University of Azores.

### Collection identifier

DTP

### Specimen preservation method

All specimens were preserved in 96% ethanol.

### Curatorial unit

Dalberto Teixeira Pombo insect collection at the University of the Azores (Curator: Paulo A. V. Borges).

## Usage licence

### Usage licence

Creative Commons Public Domain Waiver (CC-Zero)

## Data resources

### Data package title

Long-term monitoring of Azorean Forest Spiders - Part 2

### Resource link

http://ipt.gbif.pt/ipt/resource?r=spiders_azores_2021

### Alternative identifiers

https://www.gbif.org/dataset/f8b3ed49-f65d-4989-add0-9a726b1e745a

### Number of data sets

2

### Data set 1.

#### Data set name

Event Table

#### Data format

Darwin Core Archive format

#### Character set

UTF-8

#### Download URL

http://ipt.gbif.pt/ipt/resource?r=spiders_azores_2021

#### Data format version

Version 1.3

#### Description

The dataset was published in the Global Biodiversity Information Facility platform, GBIF (Lhoumeau and Borges 2022). The following data table includes all the records for which a taxonomic identification of the species was possible. The dataset submitted to GBIF is structured as a sample event dataset that has been published as a Darwin Core Archive (DwCA), which is a standardised format for sharing biodiversity data as a set of one or more data tables. The core data file contains 155 records (eventID). This GBIF IPT (Integrated Publishing Toolkit, Version 2.6.2) archives the data and, thus, serves as the data repository. The data and resource metadata are available for download in the Portuguese GBIF Portal IPT (Lhoumeau and Borges 2022).

**Data set 1.** 

Column label	Column description
id	Unique identification code for sampling event data.
eventID	Identifier of the events, unique for the dataset.
samplingProtocol	The sampling protocol used to capture the species.
sampleSizeValue	The numeric amount of time spent in each sampling.
sampleSizeUnit	The unit of the sample size value.
eventDate	Date or date range the record was collected.
eventRemarks	Information about the season and year of the event.
habitat	The habitat from which the sample was obtained.
locationID	Identifier of the location.
islandGroup	Name of archipelago.
island	Name of the island.
country	Country of the sampling site.
countryCode	ISO code of the country of the sampling site.
stateProvince	Name of the region of the sampling site.
municipality	Municipality of the sampling site.
locality	Name of the locality.
minimumElevationInMetres	The lower limit of the range of elevation (altitude, usually above sea level), in metres.
locationRemarks	Details on the locality site.
decimalLatitude	Approximate centre point decimal latitude of the field site in GPS coordinates.
decimalLongitude	Approximate centre point decimal longitude of the field site in GPS coordinates.
geodeticDatum	The ellipsoid, geodetic datum or spatial reference system (SRS) upon which the geographic coordinates given in decimalLatitude and decimalLongitude are based.
coordinateUncertaintyInMetres	Uncertainty of the coordinates of the centre of the sampling plot, in metres.
coordinatePrecision	Precision of the coordinates.
georeferenceSources	A list (concatenated and separated) of maps, gazetteers or other resources used to georeference the Location, described specifically enough to allow anyone in the future to use the same resources.

### Data set 2.

#### Data set name

Occurrence Table

#### Data format

Darwin Core Archive format

#### Character set

UTF-8

#### Download URL

http://ipt.gbif.pt/ipt/resource?r=spiders_azores_2021

#### Data format version

Version 1.3

#### Description

The dataset was published in the Global Biodiversity Information Facility platform, GBIF (Lhoumeau and Borges 2022). The following data table includes all the records for which a taxonomic identification of the species was possible. The dataset submitted to GBIF is structured as an occurrence table that has been published as a Darwin Core Archive (DwCA), which is a standardised format for sharing biodiversity data as a set of one or more data tables. The core data file contains 978 records (occurrenceID). This GBIF IPT (Integrated Publishing Toolkit, Version 2.6.2) archives the data and, thus, serves as the data repository. The data and resource metadata are available for download on the Portuguese GBIF Portal IPT (Lhoumeau and Borges 2022).

**Data set 2.** 

Column label	Column description
id	Unique identification code for species abundance data. Equivalent here to eventID.
type	Type of the record, as defined by the Public Core standard.
licence	Reference to the licence under which the record is published.
institutionID	The identity of the institution publishing the data.
collectionID	The identity of the collection publishing the data.
institutionCode	The code of the institution publishing the data.
collectionCode	The code of the collection where the specimens are conserved.
datasetName	Name of the dataset.
basisOfRecord	The nature of the data record.
recordedBy	A list (concatenated and separated) of names of people, groups or organisations who performed the sampling in the field.
occurrenceID	Identifier of the record, coded as a global unique identifier.
organismQuantity	A number or enumeration value for the quantity of organisms.
organismQuantityType	The type of quantification system used for the quantity of organisms.
sex	The sex and quantity of the individuals captured.
lifeStage	The life stage of the organisms captured.
establishmentMeans	The process of establishment of the species in the location, using a controlled vocabulary: 'native', 'introduced', 'endemic', "unknown".
eventID	Identifier of the events, unique for the dataset.
identifiedBy	A list (concatenated and separated) of names of people, groups or organisations who assigned the Taxon to the subject.
dateIdentified	The date on which the subject was determined as representing the Taxon.
scientificName	Complete scientific name including author and year.
kingdom	Kingdom name.
phylum	Phylum name.
class	Class name.
order	Order name.
family	Family name.
genus	Genus name.
specificEpithet	Specific epithet
taxonRank	Lowest taxonomic rank of the record.
scientificNameAuthorship	Name of the author of the lowest taxon rank included in the record.
identificationRemarks	Information about morphospecies identification (code in Dalberto Teixeira Pombo Collection).

## Additional information

### Results

During the 2019-2021 period, we collected a total of 5110 specimens [2449 (51%) adults], belonging to 45 species of spiders, 39 genera and 16 families. A total of fourteen species were endemic to the Azores Archipelago (2416 specimens; 1114 adults), nine species were native non-endemic (1793 specimens; 1006 adults) and twenty-two species were introduced (901 specimens, 329 adults) (Table 2).

**Figure 5.**
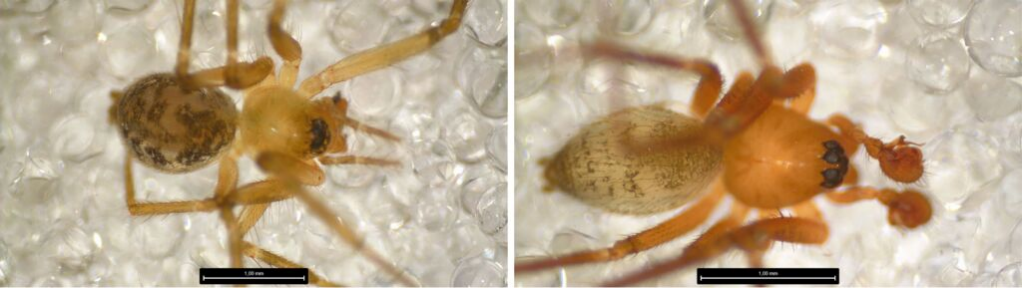
*Tenuiphantesmiguelensis* (Wunderlich, 1992), Left: Female/Right: Male, Credit: Sébastien Lhoumeau.

**Figure 6.**
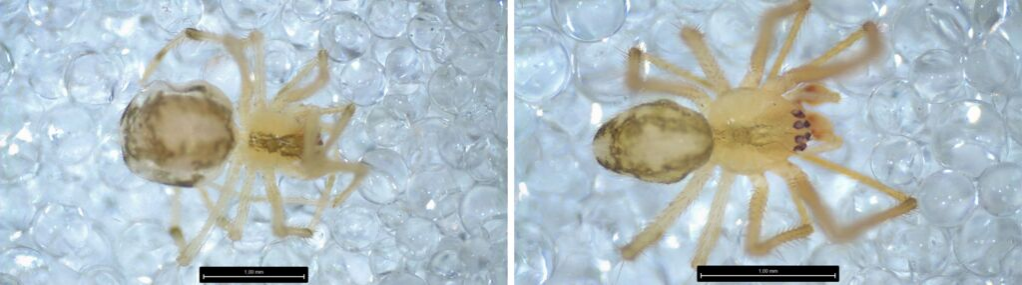
*Rugathodesacoreensis* Wunderlich, 1992, Left: Female/Right: Male, Credit: Sébastien Lhoumeau.

The ten most abundant species are composed mostly of endemic or native non-endemic species and only two exotic species (*Tenuiphantestenuis* (Blackwall, 1852) and *Dysderacrocata* C. L. Koch, 1838). The most abundant species were the endemic linyphiid *Tenuiphantesmiguelensis* with 950 specimens (776 [81%] adults) (Fig. 5) and the endemic theridiid *Rugathodesacoreensis* with 780 specimens (293 [38%] adults) (Fig. 6). Linyphiidae was the richest and most abundant family with 15 (33%) species and 2630 (51%) specimens (Table 2).

**Figure 7.**
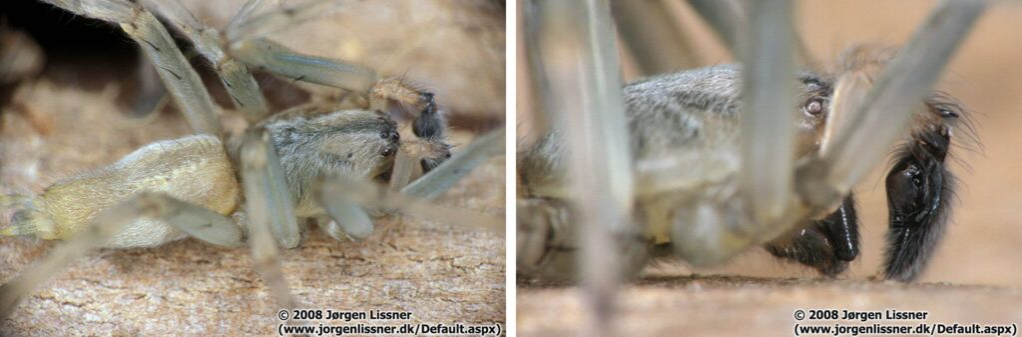
*Cheracanthiummildei* L. Koch, 1864, Left: Male Right: Detail of male pedipalp, Credit: Jørgen Lissner.

*Cheiracanthiummildei* L. Koch, 1864 is a new record for Pico Island (Fig. 7).

**Figure 8.**
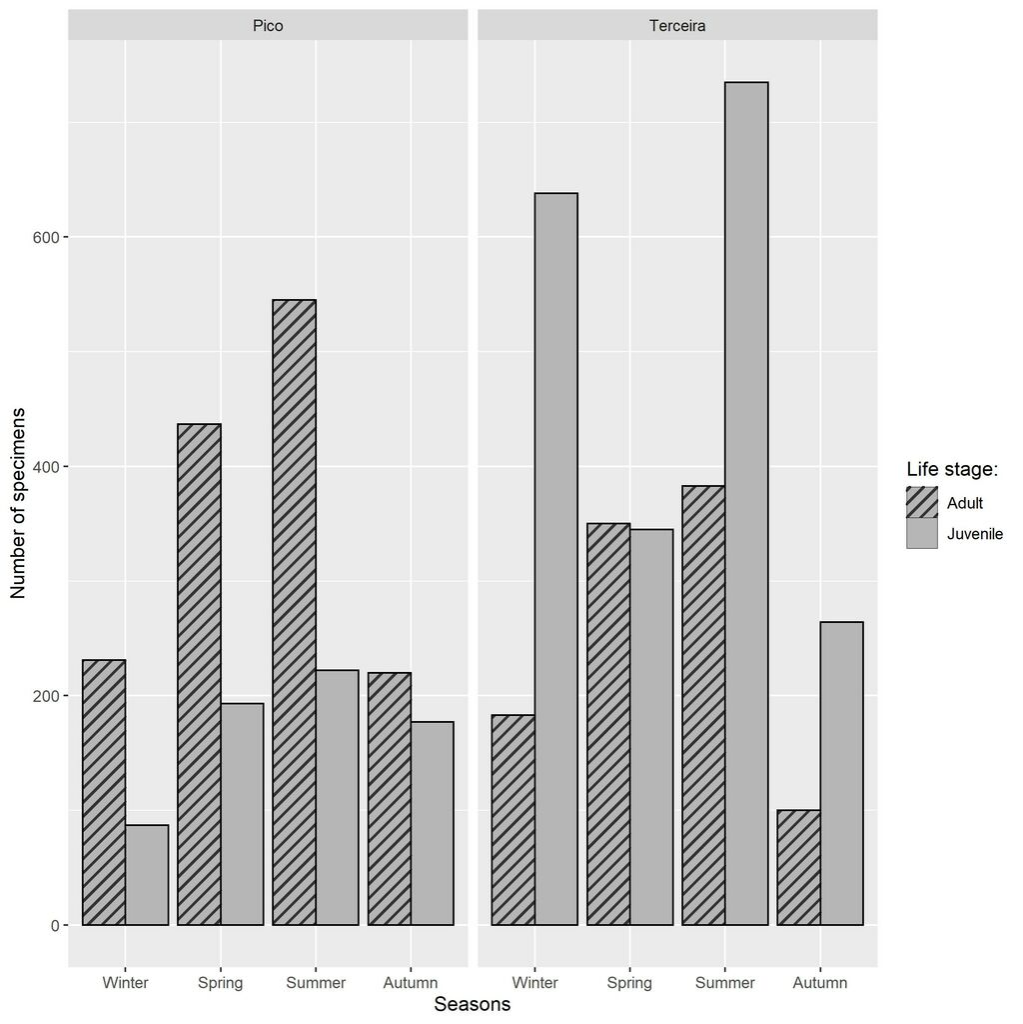
Total number of specimens collected by season during the 2019-2022 sampling period (note: from winter 2020 to autumn 2021 on Terceira and from winter 2019 to autumn 2021 in Pico).

Both on Terceira and Pico Islands, the abundance of adults gradually increased from autumn to summer, with the highest abundance occurring in summer and the lowest in autumn. On Terceira Island, we collected more juveniles than adults in all seasons, except Spring and there were two peaks of abundance in winter and in summer (Fig. 8). On Pico Island, the abundance of juveniles is lower than that of the adults for each season.

**Figure 9.**
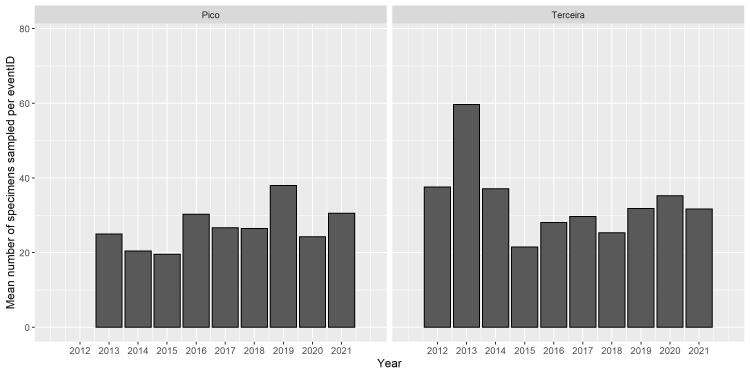
Mean number of specimens collected by year during the 2012-2021 sampling period.

We observed a slight increase in the overall abundance of the number of specimens during years of sampling, for both Islands. Such increase was more evident on Pico Island than on Terceira Island where we found a peak of abundance in 2013 followed by a drop until 2015. Finally, species abundance on both Islands is globally similar (Fig. 9). This increase can be the consequence of new exotic species sampled.

### Discussion

We analysed all available data on the two target islands – the new data collected between 2019-2021 and those from Costa and Borges (2021) – to obtain a long-term view on their forest spider assemblages. As specified previously, we considered only Event ID that were linked to only one season of sampling (about 90 days).

The use of SLAM traps for long term monitoring in native forest provides good abundance data for spiders amongst a wide variety of families (Table 3). Previous analysis of the sampling strategy revealed a sample completeness of almost 100% for the overall arthropod communities between 2013 and 2018 (Borges et al. 2020).

So far, we recorded 36 introduced, 14 endemic and nine native non-endemic species in both Terceira and Pico Islands (see Table 3). Although the indigenous/non-indigenous species ratio is in favour of introduced species, in terms of abundance, indigenous species are the most abundant group in native forests, with 75% of total specimens in Pico and 89% in Terceira.

**Figure 10.**
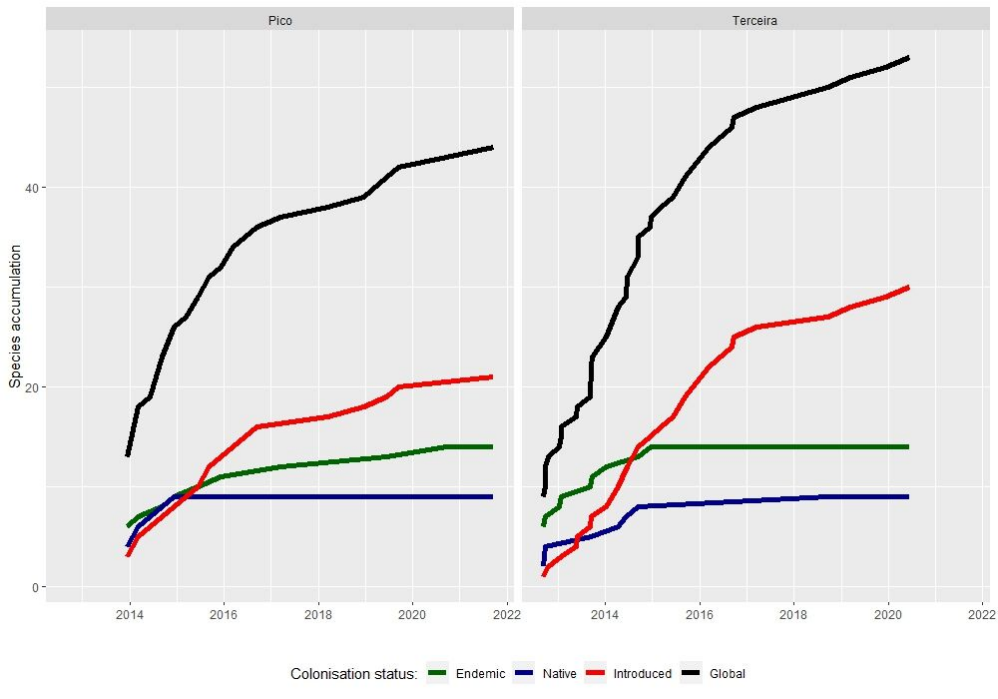
Species accumulation curves for the period 2012-2021 in the Islands of Pico and Terceira for the total species, but also for the three colonisation status groups: endemics, native-non-endemics and introduced species.

Accumulation curves computed with all our data show a global increase in the number of species through time (Fig. 10), also observed in Fig. 9. It is mainly due to the rise of introduced species. According to these curves, the majority of indigenous species were recorded in the two first years of the SLAM project, reaching an asymptote. However, the number of exotic species recorded continues to increase. It is in accordance with a recent study focusing on all arthropods across the Azores (Borges et al. 2020), which indicates that exotic species are one of the major causes of biodiversity erosion on islands (Borges et al. 2006, Borges et al. 2019). Further investigation through time series analyses is needed to assess the rate and detection of new introduced species and to properly adapt the conservation management of these areas. It is also necessary to study the temporal variation in species assemblages to detect turnover amongst exotic species that may be caused by limitations to establishment in native forest (lack of pre-adaptation, competitive exclusion, resource availability).

**Figure 11.**
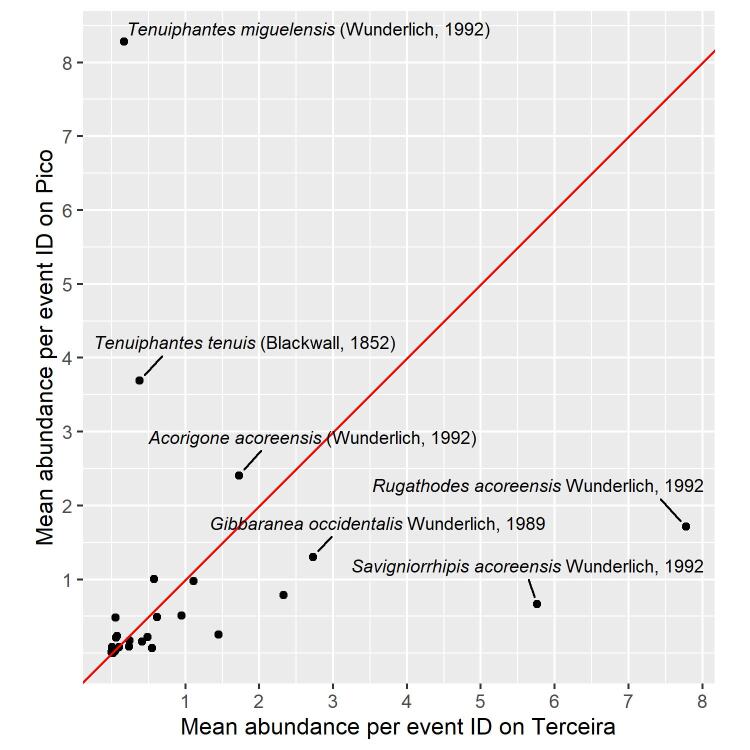
Scatter plot of mean abundance per event ID of the simultaneously collected species in Pico and Terceira Islands (n = 30 species). The red line represents the theoretical perfect match of abundance between the two Islands.

Most of the species simultaneously found on Pico and Terceira Islands share similar abundances (Fig. 11), with some exceptions. Two of the dominant species in Pico were not particularly abundant in Terceira (namely *Tenuiphantesmiguelensis* (Wunderlich, 1992) and *Tenuiphantestenuis* (Blackwall, 1852)). On the other hand, *Rugathodesacoreensis* Wunderlich, 1992 and *Savigniorrhipisacoreensis* Wunderlich, 1992 are relatively more abundant in Terceira than in Pico. The temporal dynamic of the single introduced species, *Tenuiphantestenuis*, should be monitored. These “exceptional” species and their trend through time could possibly be used as bioindicators to assess the conservation status of native forests in different Azorean Islands, since they are morphologically easily differentiable and highly abundant. This allows a quick identification, with the help of some field guides (like Vieira et al. 2021) and a rapid assessment even by non-specialist people.

Differences in the dominant species on islands might be linked to the micro-habitat preference of such species. Indeed, according to Borges and Wunderlich (2008), *Rugathodesacoreensis*, *Gibbaraneaoccidentalis* and *Savigniorrhipisacoreensis* are most common at the canopy level, while *Tenuiphantesmiguelensis* and *Tenuiphantestenuis* occur mostly at ground level. Therefore, the structure of the native forest is an important factor that may be impacting the distribution of the arachnofauna, both in terms of plant composition and architecture.

From a land use perspective, these results can be linked to the size of the native forests’ fragments. Indeed, the native forest on Pico Island is more fragmented than on Terceira Island (Borges and Hortal 2009, Triantis et al. 2010).

**Figure 12.**
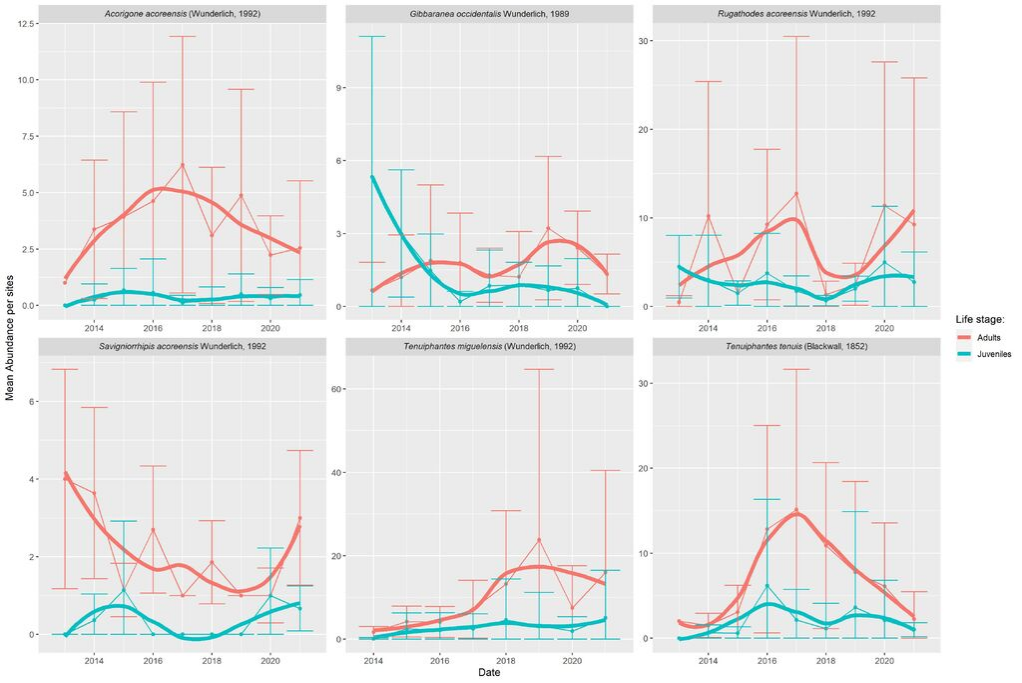
Time series of the six species showing significant differences in abundance between the two Islands (Pico Island populations).

**Figure 13.**
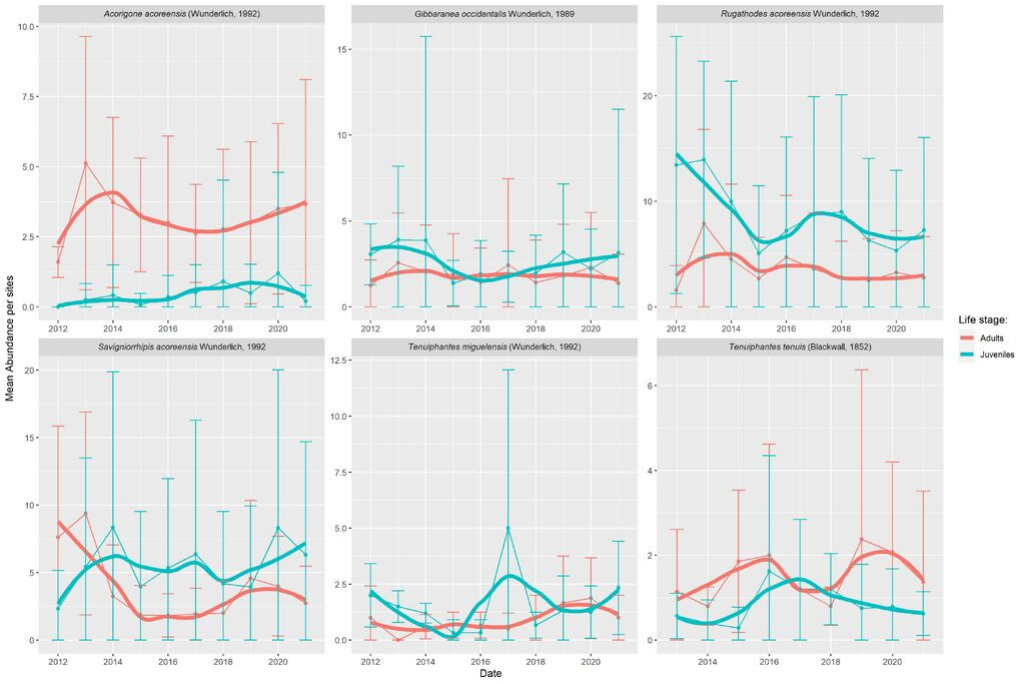
Time series of the six species showing significant differences in abundance between the two Islands (Terceira Island populations).

The majority of the most abundant species show a relatively stable abundance through time (Figs 12, 13). This stability is a positive sign of the ecosystem quality, since indigenous species are dominant in the native forests. However, more data and deeper statistical analysis are needed in future studies to confirm this apparent stability. Interestingly, *T.miguelensis* showed a slow increase of the mean number of specimens per site, but also an increase in the variation around this mean. This dynamic can be an effect of climate change (Ferreira et al. 2016) or land-use change in Pico Island (Gil et al. 2018). Finally, *Tenuiphantestenuis*, the only dominant exotic species in the dataset, exhibits a hump-shaped variation of abundance in Pico Island, where a peak of abundance was observed in 2017, when an average of circa 15 specimens per site were sampled. Its abundance is now decreasing, which is a positive observation. Particular attention should be given to this species to determine whether the trend will persist over time, as this species has already been able to successfully colonise other Macaronesia islands (Cardoso and Crespo 2008, Nuria 2010), being one of the dominant species of epigean spiders found in Madeira native forests (Boieiro et al. 2018).

The SLAM Trap sampling method is fully in accordance with the need of improving arachnofauna knowledge in terms of seasonal abundance and distribution. This kind of project should be continued to better understand the dynamic of spiders, as well as other arthropod taxa in the native forest of the Azores. Moreover, such data can also now be compared with data from other habitats like disturbed forests (Borges et al. 2022a), touristic trails (Carvalho et al. 2021), agroecosystem (Borges et al. 2021) or other disturbed habitats (Marcelino et al. 2021). The characteristics of the arachnofauna, especially species composition and abundance, can also be used to assess the habitat quality through the computation of Indices of Biotic Integrity (e.g. Cardoso et al. 2007). Additionally, these data can be useful when modelling the trends of communities through time and to prevent possible threats, mainly referring to the introduction of exotic species and extinction risk.

## Figures and Tables

**Table 1.** The list of the twenty sampled sites in the Islands of Pico (n = 7) and Terceira (n = 13).

**Island**	**Location ID**	**Site name**	**Municipality**	**Fragment name**	**Habitat**	**Latitude**	**Longitude**	**Elevation (m)**
Pico	PIC-ML-200	Plot 200m	Madalena	Mistério de St. Luzia	Mixed Forest	38.5348	-28.4341	199
Pico	PIC-ML-400	Plot 400m	Madalena	Mistério de St. Luzia	Mixed Forest	38.5207	-28.4311	428
Pico	PIC-ML-600	Plot 600m	São Roque do Pico	Mistério de St. Luzia	Mixed Forest	38.5119	-28.4189	627
Pico	PIC-ML-800	Plot 800m	São Roque do Pico	Mistério de St. Luzia	Mixed Forest	38.4999	-28.4229	797
Pico	PIC-NFCA-T-09	Caveiro Base	Lajes do Pico	Pico Caveiro	Native Forest	38.4377	-28.2106	937
Pico	PIC-NFLC-T-02	Euphorbias	Lajes do Pico	Lagoa do Caiado	Native Forest	38.4561	-28.2577	804
Pico	PIC-NFMP-T-03	Chão Verde inferior	São Roque do Pico	Mistério da Prainha	Native Forest	38.4876	-28.2733	475
Terceira	TER-0M	Farol da Serreta	Angra do Heroísmo	Farol da Serreta	Erica Forest	38.7666	-27.3748	46
Terceira	TER-200M	Serreta 200m	Angra do Heroísmo	Mata da Serreta	Mixed Forest	38.7604	-27.3638	237
Terceira	TER-400M	Mirador do Pico Carneiro	Angra do Heroísmo	Mata da Serreta	Mixed Forest	38.7621	-27.3476	397
Terceira	TER-NFBF-T-01	Labaçal -Morro Assombrado	Praia da Vitória	Biscoito da Ferraria	Native Forest	38.7618	-27.2193	678
Terceira	TER-NFBF-T-02	Chambre A	Praia da Vitória	Biscoito da Ferraria	Native Forest	38.7521	-27.2331	590
Terceira	TER-NFBF-TP41	Pico Alto Nascente	Praia da Vitória	Biscoito da Ferraria	Native Forest	38.7502	-27.2072	673
Terceira	TER-NFPG-T-33	Pico X B	Praia da Vitória	Pico Galhardo	Native Forest	38.7334	-27.2271	642
Terceira	TER-NFSB-T-07	Lomba	Angra do Heroísmo	Serra de Santa Bárbara	Native Forest	38.7372	-27.2899	683
Terceira	TER-NFSB-T164	Caldeira - Silvia	Angra do Heroísmo	Serra de Santa Bárbara	Native Forest	38.7355	-27.3074	900
Terceira	TER-NFSB-TE48	Lagoinha B	Angra do Heroísmo	Serra de Santa Bárbara	Native Forest	38.7521	-27.3313	687
Terceira	TER-NFSB-TE49	Lagoa Pinheiro B	Angra do Heroísmo	Serra de Santa Bárbara	Native Forest	38.7471	-27.3196	918
Terceira	TER-NFTB-T-15	Terra Brava -A	Praia da Vitória	Terra Brava	Native Forest	38.7364	-27.2006	637
Terceira	TER-NFTB-T-18_ORIGINAL	Terra Brava -B -Original	Praia da Vitória	Terra Brava	Native Forest	38.7323	-27.1980	686

**Table 2.** List of the recorded species between 2019 and 2021 with their corresponding family, colonisation status (E - endemic from Azores; N - native non-endemic; I - exotic introduced species), IUCN status for the endemic species (in parenthesis together with the colonisation status; VU - Vulnerable; NT - Near Threatened; LC - Least Concern) and overall abundance (adults + juveniles) in each of the two studied Islands and total abundance of specimens. The new records are marked with a (*). The ten most abundant species are in bold.

**Family**	**Species**	**Colonis.**	**Pico**	**Terceira**	**Total** **Abundance**
Agelenidae	*Tegenariadomestica* (Clerck, 1757)	I	2	0	2
Agelenidae	*Textrixcaudata* L. Koch, 1872	I	0	5	5
Araneidae	***Gibbaraneaoccidentalis* Wunderlich, 1989**	E (NT)	81	265	346
Cheiracanthiidae	*Cheiracanthiumerraticum* (Walckenaer, 1802)	I	9	8	17
Cheiracanthiidae	*Cheiracanthiummildei* L. Koch, 1864	I	11(*)	0	11
Clubionidae	*Clubionaterrestris* Westring, 1851	I	23	2	25
Clubionidae	*Porrhoclubionadecora* (Blackwall, 1859)	N	25	19	44
Clubionidae	*Porrhoclubionagenevensis* (L. Koch, 1866)	I	18	20	38
Dictynidae	*Emblynaacoreensis* Wunderlich, 1992	E (NT)	5	1	6
Dictynidae	***Lathysdentichelis* (Simon, 1883)**	N	61	244	305
Dictynidae	*Nigmapuella* (Simon, 1870)	I	8	1	9
Dysderidae	***Dysderacrocata* C. L. Koch, 1838**	I	108	79	187
Linyphiidae	***Acorigoneacoreensis* (Wunderlich, 1992)**	E (VU)	152	200	352
Linyphiidae	*Canariphantesacoreensis* (Wunderlich, 1992)	E (VU)	22	18	40
Linyphiidae	*Erigoneatra* Blackwall, 1833	I	1	1	2
Linyphiidae	*Erigoneautumnalis* Emerton, 1882	I	0	2	2
Linyphiidae	***Microlinyphiajohnsoni* (Blackwall, 1859)**	N	117	160	277
Linyphiidae	*Miniciafloresensis* Wunderlich, 1992	E (VU)	2	9	11
Linyphiidae	*Nerieneclathrata* (Sundevall, 1830)	I	1	0	1
Linyphiidae	*Palliduphantesschmitzi* (Kulczynski, 1899)	N	4	0	4
Linyphiidae	*Pelecopsisparallela* (Wider, 1834)	I	0	1	1
Linyphiidae	*Porrhommaborgesi* Wunderlich, 2008	E (VU)	4	1	5
Linyphiidae	*Prinerigonevagans* (Audouin, 1826)	I	0	1	1
Linyphiidae	***Savigniorrhipisacoreensis* Wunderlich, 1992**	E (VU)	25	607	632
Linyphiidae	***Tenuiphantesmiguelensis* (Wunderlich, 1992)**	N	912	38	950
Linyphiidae	***Tenuiphantestenuis* (Blackwall, 1852)**	I	243	57	300
Linyphiidae	*Walckenaeriagrandis* (Wunderlich, 1992)	E (VU)	7	45	52
Lycosidae	*Pardosaacorensis* Simon, 1883	E (LC)	1	0	1
Mimetidae	*Erofurcata* (Villers, 1789)	I	7	86	93
Pisauridae	*Pisauraacoreensis* Wunderlich, 1992	E (NT)	9	25	34
Salticidae	***Macaroeriscata* (Blackwall, 1867)**	N	19	160	179
Salticidae	*Macaroerisdiligens* (Blackwall, 1867)	N	1	7	8
Salticidae	*Neonacoreensis* Wunderlich, 2008	E (VU)	1	0	1
Segestriidae	*Segestriaflorentina* (Rossi, 1790)	I	0	1	1
Tetragnathidae	*Metellinamerianae* (Scopoli, 1763)	I	8	6	14
Tetragnathidae	*Leucognathaacoreensis* (Wunderlich, 1992)	E (VU)	20	134	154
Theridiidae	*Cryptachaeablattea* (Urquhart, 1886)	I	27	121	148
Theridiidae	*Lasaeolaoceanica* Simon, 1883	E (LC)	0	2	2
Theridiidae	*Parasteatodatepidariorum* (C. L. Koch, 1841)	I	0	20	20
Theridiidae	***Rugathodesacoreensis* Wunderlich, 1992**	E (NT)	148	632	780
Theridiidae	*Steatodagrossa* (C. L. Koch, 1838)	I	3	3	6
Theridiidae	*Steatodanobilis* (Thorell, 1875)	I	7	9	16
Theridiidae	*Theridionmusivivum* Schmidt, 1956	N	0	2	2
Thomisidae	*Xysticuscor* Canestrini, 1873	N	18	6	24
Zoropsidae	*Zoropsisspinimana* (Dufour, 1820)	I	2	0	2

**Table 3.** The list of all species sampled between 2012 and 2022, mentioning the family, colonisation status (E - endemic from Azores; N - native non-endemic; I - exotic introduced species), IUCN status for the endemic species (VU - Vulnerable; NT - Near Threatened; LC - Least Concern) indication of overall abundance (adults + juveniles) in the two studied islands and total abundance. The ten most abundant species are in bold.

**Family**	**Species**	**Colonis.**	**Pico**	**Terceira**	**Grand total**
Agelenidae	*Tegenariadomestica* (Clerck, 1757)	I	2	1	3
Agelenidae	*Tegenariapagana* C. L. Koch, 1840	I	0	1	1
Agelenidae	*Textrixcaudata* L. Koch, 1872	I	1	42	43
Araneidae	*Agalenatearedii* (Scopoli, 1763)	I	0	2	2
Araneidae	*Araneusangulatus* Clerck, 1757	I	0	1	1
Araneidae	***Gibbaraneaoccidentalis* Wunderlich, 1989**	E (NT)	273	1330	1603
Araneidae	*Mangoraacalypha* (Walckenaer, 1802)	I	1	0	1
Araneidae	*Zygiellax-notata* (Clerck, 1757)	I	6	0	6
Cheiracanthiidae	*Cheiracanthiumerraticum* (Walckenaer, 1802)	I	14	40	54
Cheiracanthiidae	*Cheiracanthiummildei* L. Koch, 1864	I	11	0	11
Clubionidae	*Clubionaterrestris* Westring, 1851	I	82	3	85
Clubionidae	*Porrhoclubionadecora* (Blackwall, 1859)	N	102	268	370
Clubionidae	*Porrhoclubionagenevensis* (L. Koch, 1866)	I	27	45	72
Dictynidae	*Emblynaacoreensis* Wunderlich, 1992	E (NT)	7	6	13
Dictynidae	***Lathysdentichelis* (Simon, 1883)**	N	167	1140	1307
Dictynidae	*Nigmapuella* (Simon, 1870)	I	12	11	23
Dysderidae	*Dysderacrocata* C. L. Koch, 1838	I	213	276	489
Linyphiidae	***Acorigoneacoreensis* (Wunderlich, 1992)**	E (VU)	506	836	1342
Linyphiidae	*Agynetadecora* (O. Pickard-Cambridge, 1871)	I	0	4	4
Linyphiidae	*Canariphantesacoreensis* (Wunderlich, 1992)	E (VU)	46	42	88
Linyphiidae	*Entelecaraschmitzi* Kulczynski, 1905	I	0	11	11
Linyphiidae	*Erigoneatra* Blackwall, 1833	I	1	10	11
Linyphiidae	*Erigoneautumnalis* Emerton, 1882	I	0	3	3
Linyphiidae	*Erigonedentipalpis* (Wider, 1834)	I	0	5	5
Linyphiidae	*Mermessusfradeorum* (Berland, 1932)	I	1	0	1
Linyphiidae	***Microlinyphiajohnsoni* (Blackwall, 1859)**	N	203	616	819
Linyphiidae	*Miniciafloresensis* Wunderlich, 1992	E (VU)	4	22	26
Linyphiidae	*Nerieneclathrata* (Sundevall, 1830)	I	3	0	3
Linyphiidae	*Oedothoraxfuscus* (Blackwall, 1834)	I	1	3	4
Linyphiidae	*Palliduphantesschmitzi* (Kulczynski, 1899)	N	27	5	32
Linyphiidae	*Pelecopsisparallela* (Wider, 1834)	I	0	11	11
Linyphiidae	*Porrhommaborgesi* Wunderlich, 2008	E (VU)	4	6	10
Linyphiidae	*Prinerigonevagans* (Audouin, 1826)	I	0	1	1
Linyphiidae	***Savigniorrhipisacoreensis* Wunderlich, 1992**	E (VU)	138	2983	3121
Linyphiidae	***Tenuiphantesmiguelensis* (Wunderlich, 1992)**	N	1730	92	1822
Linyphiidae	***Tenuiphantestenuis* (Blackwall, 1852)**	I	768	167	935
Linyphiidae	*Walckenaeriagrandis* (Wunderlich, 1992)	E (VU)	46	308	354
Lycosidae	*Arctosaperita* (Latreille, 1799)	I	0	2	2
Lycosidae	*Pardosaacorensis* Simon, 1883	E (LC)	8	20	28
Mimetidae	*Erofurcata* (Villers, 1789)	I	14	505	519
Pholcidae	*Pholcusphalangioides* (Fuesslin, 1775)	I	0	3	3
Pisauridae	*Pisauraacoreensis* Wunderlich, 1992	E (NT)	35	126	161
Salticidae	***Macaroeriscata* (Blackwall, 1867)**	N	53	688	741
Salticidae	*Macaroerisdiligens* (Blackwall, 1867)	N	6	41	47
Salticidae	*Neonacoreensis* Wunderlich, 2008	E (VU)	1	5	6
Salticidae	*Pseudeuophrysvafra* (Blackwall, 1867)	I	0	8	8
Salticidae	*Salticusmutabilis* Lucas, 1846	I	0	6	6
Segestriidae	*Segestriaflorentina* (Rossi, 1790)	I	0	5	5
Tetragnathidae	*Metellinamerianae* (Scopoli, 1763)	I	13	17	30
Tetragnathidae	***Leucognathaacoreensis* (Wunderlich, 1992)**	E (VU)	126	728	854
Theridiidae	*Cryptachaeablattea* (Urquhart, 1886)	I	34	325	359
Theridiidae	*Lasaeolaoceanica* Simon, 1883	E (LC)	5	12	17
Theridiidae	*Parasteatodatepidariorum* (C. L. Koch, 1841)	I	0	28	28
Theridiidae	***Rugathodesacoreensis* Wunderlich, 1992**	E (NT)	358	3969	4327
Theridiidae	*Steatodagrossa* (C. L. Koch, 1838)	I	4	6	10
Theridiidae	*Steatodanobilis* (Thorell, 1875)	I	100	25	125
Theridiidae	*Theridionmusivivum* Schmidt, 1956	N	17	3	20
Thomisidae	*Xysticuscor* Canestrini, 1873	N	48	38	86
Zoropsidae	*Zoropsisspinimana* (Dufour, 1820)	I	20	0	20
	**GRAND TOTAL**		**5238**	**14851**	**20089**
